# A Smartphone-Based Automatic Measurement Method for Colorimetric pH Detection Using a Color Adaptation Algorithm

**DOI:** 10.3390/s17071604

**Published:** 2017-07-10

**Authors:** Sung Deuk Kim, Youngmi Koo, Yeoheung Yun

**Affiliations:** 1 Department of Electronic Engineering Education, Andong National University, 1375 Gyeongdong-ro, Andong, Gyeongsangbuk-do 36729, Korea; sdkim@andong.ac.kr; 2 FIT BEST Laboratory, Department of Chemical, Biological, and Bioengineering, North Carolina A&T State University, 1601 E. Market St., Greensboro, NC 27411, USA; kooym20120503@gmail.com

**Keywords:** colorimetric sensor measurement, pH detection, smartphone-based measurement

## Abstract

This paper proposes a smartphone-based colorimetric pH detection method using a color adaptation algorithm for point-of-care applications. Although a smartphone camera can be utilized to measure the color information of colorimetric sensors, ambient light changes and unknown built-in automatic image correction operations make it difficult to obtain stable color information. This paper utilizes a 3D printed mini light box and performs a calibration procedure with a paper-printed comparison chart and a reference image which overcomes the drawbacks of smartphone cameras and the difficulty in preparing for the calibration procedure. The color adaptation is performed in the CIE 1976 u’v’ color space by using the reference paper in order to stabilize the color variations. Non-rigid u’v’ curve interpolation is used to produce the high-resolution pH estimate. The final pH value is estimated by using the best-matching method to handle the nonlinear curve properties of multiple color patches. The experimental results obtained using a pH indicator paper show that the proposed algorithm provides reasonably good estimation of pH detection. With paper-printed accurate color comparison charts and smart color adaptation techniques, superior estimation is achieved in the smartphone-based colorimetric pH detection system for point-of-care application.

## 1. Introduction

Colorimetric microfluidic paper-based analytical devices (µPADs) are emerging as new point-of-care devices, which have advantages of low-cost manufacturing, easy disposability, as well as the fact no external power is needed for fluidic transportation [1]. Traditional colorimetric-based tests such as pregnancy tests depend on true or false result determined by the naked eye and cannot provide quantified values since they have poor accuracy that is sensitive to the environment, and need an expensive camera. After smartphones were developed and became widespread, there have been many studies to utilize smartphones as digital instruments for water quality monitoring [2,3,4], health-related diagnostics [5,6], and various special-purpose colorimetric sensor measurements [7,8,9,10]. In particular, smartphone-based pH measurements have been used for various applications [4,11,12]. Smartphone cameras are cheap, portable, and easy to use, however they have some drawbacks. They are vulnerable to ambient light changes. In addition, their unknown built-in automatic image correction operations such as automatic brightness control and white balance prevent a smartphone camera from acquiring stable color information of colorimetric sensors [11]. Therefore, they require additional image calibration techniques for acquiring color information in a stable way. Chang used the hue component only in colorimetric measurements for handling the brightness variation [12]. Shen et al. proposed a point-of-care colorimetric detection method in which a pH strip image is acquired with a known reference image and a two-dimensional calibration plane is applied in the CIE 1931 xy color space to stabilize the built-in camera functions [11]. Schaefer proposed two colorimetric water quality detection systems using smartphones with additional hardware modules [4]. The first system attaching a sensor module to the smartphone uses the built-in flash as the light source and the smartphone camera measures the water quality as the pH value. The second system uses a separate color sensor and light source to measure the water quality, and the smartphone is used for user interface only via wireless connection.

Colors are determined by light sources and image sensors. Many colorimetric sensor measurement studies have focused on finding the true color of a material by isolating the side effects that may be caused by ambient light and intrinsic image sensor properties. In order to cope with the ambient light effect, which is not a simple matter, we implement a mini light box incorporating two LED light modules to isolate the ambient light effect. 3D Printers have recently been used extensively to implement analytical sensing devices [2,4,7], so we built a simple mini light box model with a 3D printer. A light box accessory attached to a fixed smartphone model makes it easy for a developer to build the system accurately. However, the type of smartphone model that can be used is limited on the user side. We choose a simple mini light box structure that allows a user to manually place a smartphone on the mini light box, even though it requires an additional automatic color retrieving algorithm.

In order to cope with various intrinsic image sensor properties, calibration techniques [13] are usually applied with real accurate pH buffers or high-quality verified pH measuring devices [4]. However, just like the typical use of pH paper that does not require real accurate pH buffers, we use the paper-printed color comparison chart provided by the pH indicator paper manufacturer and the reference image composed of various colors to perform the color calibration in the proposed system. The calibration step is based on color adaptation in the CIE 1976 u’v’ color space [14] and non-rigid u’v’ curve interpolation is used to obtain high-precision pH estimate. In the measurement step, a best-matching method handling the nonlinear curve properties of multiple color patches is utilized instead of deriving a specific analytic estimation function based on linear fitting or curve fitting.

In our system, the smartphone camera is not a fixed non-mobile instrument device with common camera attributes. The relative positions of the color patches in the captured image are changeable for various reasons, such as various camera aspect ratios, focal lengths, and the location of the smartphone camera. Therefore, an automatic color retrieving algorithm based on contour extraction is also proposed to automatically obtain all the color values of the captured image.

Three Android smartphones and Whatman pH indicator paper are used in the experiments to evaluate the performance of the developed colorimetric pH detection system. After checking whether the color information is retrieved automatically from the captured image, we examine the detailed results of the calibration step and the measurement step.

## 2. Materials and Methods 

### 2.1. Materials

In this paper, we utilize a 3D printed mini light box, a pH indicator paper, a reference paper, three Android smart phones, and pH buffer solutions. The 3D printed mini light box is modeled by OpenSCAD [15], a script-based 3D object modeling software, and it is printed with black resin by a Formlabs Form 1+ 3D printer [16]. Two white LED backlight modules used as the light source in the mini light box were purchased from Adafruit [17]. Whatman pH indicator paper with resolution of 1.0 (pH 0~14, No. 2613991 from Fisher Scientific, Waltham, MA, USA) was used. Each strip of the pH indicator paper has four colorimetric sensor patches. A reference paper used for color adaptation algorithm is printed with a photo printer. Three Android smartphones (version 4.x) manufactured by Samsung (Suwon, Korea) and LG Electronics (Seoul, Korea) were used. An Android App was developed for camera image capturing and pH measurement. All images are captured after all camera settings are set to ‘auto’. The calibration algorithm implemented with C++ and Octave [18] language is performed offline on a personal computer after the calibration-purpose captured images are obtained by the smartphone. The core of measurement algorithm that is purely programmed with C++ language is implemented in the Android App via Android Native Development Kit (NDK) [19]. The OpenCV software library is utilized for implementing the image processing parts [20]. In order to evaluate the pH detection performance, real samples of standard pH buffer solutions and a few other samples of phosphate buffer solutions (BS) and phosphate-buffered saline (PBS) are used. pH buffer solutions of pH 1.68, 4.01, 7.00, 10.01, 12.46 (No. 916099, 910168, 910112, Orion, Waltham, MA, USA), pH 6.0 BS (Ricca, Arlington, TX, USA), pH 6.5 BS (EMS, Hatfield, PA, USA), and pH 7.4 PBS (Gibco, Waltham, MA, USA) were used for verification.

### 2.2. Calibration Step Using a Paper-Printed Comparison Chart

From an ideal point of view, the calibration step is to establish an analytic relationship between the color information of the pH strip and the ideal pH values by examining accurate known pH buffers. Once the calibration step is completed, the pH value can be estimated from the color information of the pH strip to be measured. In this paper, however we utilize the paper-printed comparison chart image that is usually supplied together with pH strips instead of using accurate known pH buffers in the calibration step. This approach is attractive because a user doesn’t need accurate calibration-purpose pH buffers. Typical use of pH strips doesn’t require for a user to buy accurate calibration-purpose pH buffers. A user simply compares the pH strip with the comparison chart and finds the best-match to estimate the pH value. We follow the basic concept of the typical pH strip usage in the calibration and measurement steps. However, since the measured color can vary due to a variety of factors caused by smartphone camera characteristics such as the unknown built-in automatic color control mechanism, the measured color should be normalized by a color adaptation algorithm. The color adaptation is performed by utilizing the reference images captured together with a pH strip. The color adaptation makes the color of reference images be the same and eventually produces the calibrated color information even if the camera characteristics are changed.

**Figure 1:**
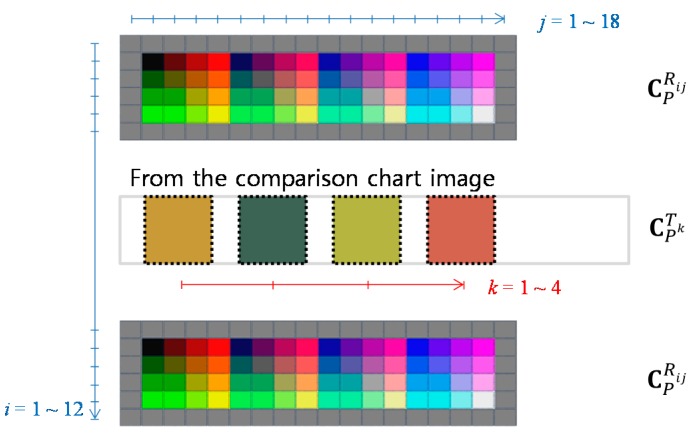
Notations for color information obtained for calibration.

All color information on the reference regions composed of various colors and the target region where the paper-printed pH strip is placed are collected automatically. Here, the obtained colors are depicted in Figure 1 to explain the following calibration procedure. In Figure 1, the colors of the upper and lower reference regions for calibration are noted as $C_{P}^{R_{ij}}$ and the colors of the target region for calibration are noted as $C_{P}^{T_{k}}$, where *P* is the pH index, *i* and *j* are the reference color indexes, and *k* is the target color index. In this paper, *P* = 0~14, *i* = 1~12, *j* = 1~18, and *k* = 1~4. Therefore, total number of indexes for the reference image is 216 (=12 × 18).

We perform a color adaptation method in the CIE 1976 u’v’ color space. One of the pH index values is chosen as the pivot index noted as Φ. We derive a color converting model to match the reference color of the captured image with the reference color of the pivot index image. Although various color adaptation algorithms such as the linear von Kries based adaptation model can be applied [21], we use the third-order polynomial model of 20 parameters expressed as in Equations (1) and (2) for color adaptation.

(1) $$\left[\begin{array}{c} u_{d} \end{array}\right]=\left[\begin{array}{cccccccccc} u_{s} & v_{s} & 1 & u_{s}^{2} & v_{s}^{2} & u_{s}v_{s} & u_{s}^{3} & v_{s}^{3} & u_{s}^{2}v_{s} & u_{s}v_{s}^{2} \end{array}\right]\left[\begin{array}{c} a_{00}\\ a_{01}\\ \vdots \\ a_{09} \end{array}\right]$$

(2) $$\left[\begin{array}{c} v_{d} \end{array}\right]=\left[\begin{array}{cccccccccc} u_{s} & v_{s} & 1 & u_{s}^{2} & v_{s}^{2} & u_{s}v_{s} & u_{s}^{3} & v_{s}^{3} & u_{s}^{2}v_{s} & u_{s}v_{s}^{2} \end{array}\right]\left[\begin{array}{c} a_{10}\\ a_{11}\\ \vdots \\ a_{19} \end{array}\right]$$

where $\left(u_{s},v_{s}\right)$ and $\left(u_{d},v_{d}\right)$ are u’v’ color components of $C_{P}^{R_{ij}}$ and $C_{\Phi }^{R_{ij}}$, respectively. $a_{00}\;\sim \;a_{09}$ and $a_{10}\;\sim \;a_{19}$ are the 20 parameters. For each pH index *P*, the color converting model is derived and denoted as $F_{P}$. In order to obtain a robust estimate, we use the minimum absolute deviation solution obtained by iterative reweighted least square [22]. Since the color information $\left(u_{s},v_{s}\right)$ and $\left(u_{d},v_{d}\right)$ of all reference color indexes can be expressed in a single matrix form as y=Xa by considering Equations (1) or (2), the optimal solution a is formulated as Equation (3). Here, $arg\;\underset{a}{\min}C\left(a\right)$ means the argument that minimizes the cost function C(a):

(3) $$arg\;\underset{a}{\min}{\left||y-Xa|\right|}_{1}=arg\;\underset{a}{\min}\overset{216}{\underset{i=1}{\sum }}\left|y_{i}-X_{i}a\right|$$

The solution a is obtained by iterative reweighted least square as expressed in Equation (4).

(4) $$a^{\left(t+1\right)}={\left(X^{T}W^{\left(t\right)}X\right)}^{-1}X^{T}W^{\left(t\right)}y$$

where $W^{\left(t\right)}$ is the diagonal matrix of weights of Equations (5) and (6).

(5) $$w_{i}^{\left(0\right)}=1$$

(6) $$w_{i}^{\left(t\right)}=\frac{1}{\max\left(\delta ,\left|y_{i}-X_{i}a^{\left(t\right)}\right|\right)}\;\left(t>0\right)$$

In this paper, δ is set to 0.01 and the number of iterations is 20. The pivot index Φ is set to 7. 

After the color converting model $F_{P}$ is derived, the initial target color $C_{P}^{T_{k}}$ is converted to the calibrated target color ${\mathbb{C}}_{P}^{T_{k}}$ by using the same color converting model $F_{P}$. 

In order to achieve better resolution of pH estimate from ${\mathbb{C}}_{P}^{T_{k}}$, we apply thin plate spline interpolation [23,24] and obtain the high-resolution calibrated target colors. The interpolation is achieved by finding $f_{k}\left(x\right)$ that minimizes the energy function of Equation (7):

(7) $$E\left(f_{k}\right)=E_{error}\left(f_{k}\right)+\lambda E_{smooth}\left(f_{k}\right)=\overset{14}{\underset{P=0}{\sum }}{\left||{\mathbb{C}}_{P}^{T_{k}}-f_{k}\left(P\right)|\right|}^{2}+\lambda \int \left[{\left(f_{k}\prime \prime \right)}^{2}\right]dx$$

In Equation (7), the tuning parameter 𝜆 is used to control how non-rigidness is allowed for the deformation. In this paper, 𝜆 is set to 3/7 so that the energy ratio of $E_{error}\left(f_{k}\right)$:$E_{smooth}\left(f_{k}\right)$ becomes 7:3. If 𝜆 is 0, only $E_{error}\left(f_{k}\right)$ is used; if 𝜆 is ∞, only $E_{smooth}\left(f_{k}\right)$ is used and the estimated curve becomes a straight line. The high-resolution calibrated target colors ${𝕔}_{p}^{T_{k}}$ are obtained by $f_{k}\left(x\right)$. In this paper, the pH resolution of 0.1 is used from pH ≈ 0.0 to pH ≈ 14.0 for ${𝕔}_{p}^{T_{k}}$.

It should be noted that the final calibration data to be stored for the following measurement procedure are the reference colors of the pivot index for calibration, $C_{\Phi }^{R_{ij}}$ , and the high-resolution calibrated target colors, ${𝕔}_{p}^{T_{k}}$, where *p* = 0.0, 0.1, 0.2,…, 14.0. 

### 2.3. Measurement Step Based on Color Adaptation and Best-Matching

All colors in the reference regions and the target region are obtained in a similar way as in the calibration step. However, we denote the reference colors and the target colors obtained in the measurement step as $M^{R_{ij}}$ and $M^{T_{k}}$, respectively.

In the measurement step, color adaptation is performed between $M^{R_{ij}}$ and $C_{\Phi }^{R_{ij}}$ in a similar way as performed in the calibration step based on the color adaptation model of Equations (1) and (2). However, in the measurement step, $\left(u_{s},v_{s}\right)$ and $\left(u_{d},v_{d}\right)$ are u’v’ color components of $M^{R_{ij}}$ and $C_{\Phi }^{R_{ij}}$, respectively. The color converting model derived in the measurement step is denoted as $F_{M}$, and the initial target color $M^{T_{k}}$ is converted to the calibrated target color $M^{T_{k}}$ by using the same color converting model $F_{M}$.

The final pH estimate is obtained by finding the best match of $M^{T_{k}}$ among ${𝕔}_{p}^{T_{k}}$ as expressed in Equation (8):

(8) $$arg\;\underset{p}{\min}\overset{4}{\underset{k=1}{\sum }}{\left||M^{T_{k}}-{𝕔}_{p}^{T_{k}}|\right|}_{1}$$

Instead of deriving a linear equation or performing curve fitting, this approach doesn’t require any prior knowledge of best-fitting curves and it can be used for pH strips with multiple color patches. In this paper, we used the pH strip composed of four color patches. 

### 2.4. Automatic Color Retrieving Algorithm

Since the smartphone cameras with different characteristics are manually placed on the mini light box, the color patch positions in the captured image are not fixed. Therefore, in order to retrieve the color information for calibration and measurement steps, it is necessary to find all color patch positions in the captured image automatically. The proposed procedure of the automatic color retrieving algorithm based on contour extraction consists of the following six steps: first, a noise reduction and image simplification step is performed on the input captured image. Second, the edges of the simplified image are detected. Third, the initial contours are extracted with the initial constraints that accept only rectangular shapes and ignore small size contours and non-convex contours. Fourth, the final contour labeling step follows. In this step, the contours of the reference region and the target region are separated. The reference region constraints accept only the rectangles whose aspect ratio is greater than 3.5 because the reference paper of 30 × 8 mm (aspect ratio = 3.75) is used in this experiment. It also rejects the contours near center line because the contours near the center line tend to belong to the target region. In a similar way, the target region constraints accept only the squares near center line and reject the contours of different size. After the sorting and labeling steps, the final contours for the reference region and the target region are determined. Fifth, the positions for retrieving the color information of the upper reference region are determined. Principal component analysis is performed to obtain principal axes of the upper reference rectangle contour. And then four crossing points between the principal axes and the upper reference rectangle are found. 6 × 18 upper reference region positions are determined using the crossing points. Similar approach is also applied for lower reference region. After the positions of the reference colors are obtained, the positions for the target colors are calculated by using the averaged center and slope of the two reference regions. Finally, the reference colors and the target colors are retrieved from the determined positions. 

## 3. Results and Discussion

### 3.1. Automatic Color Retrieving in the 3D Printed Mini Light Box

**Figure 2:**
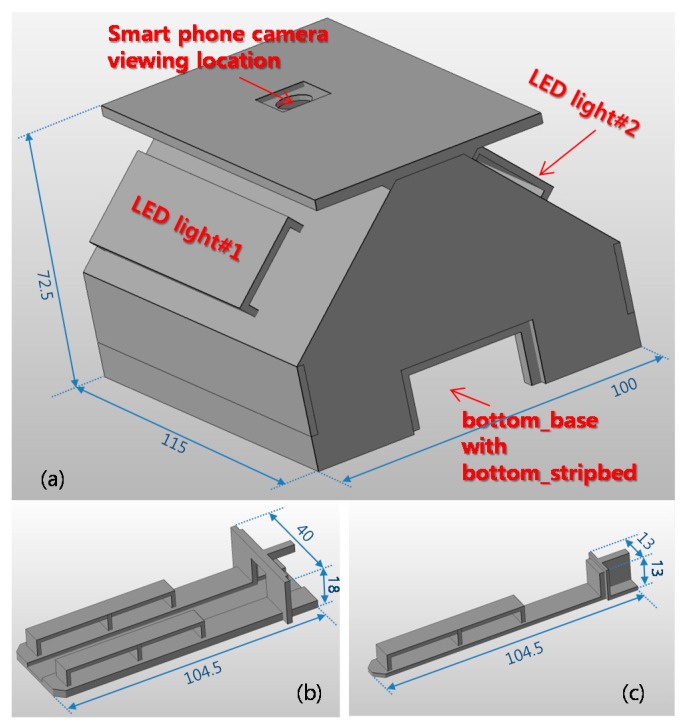
3D printed mini light box: (**a**) Body; (**b**) Bottom_base; (**c**) Bottom_stripbed. The implemented size is displayed in mm.

One of the important things in the process of measuring colorimetric sensor is isolating ambient light effects. Ambient light conditions are so complex that simple software algorithms cannot isolate them effectively. In this experiment, we have built a 3D printed mini light box consisting of three parts; body, bottom_base, and bottom_stripbed as shown in Figure 2. The body isolates ambient light and provides a light source. Two LED lighting modules are used as the light source. Each LED is connected to a 5 V supply through a 220 Ω current limiting resistor. Smartphone is placed on top of the body and its camera is located over the center hole on the body. On the two top planes of the bottom_base, the reference images composed of various colors are placed for the purpose of calibration. The pH strip to measure is placed on top plane of the bottom_stripbed. The bottom_stripbed is inserted into the bottom_base and the bottom_base is inserted into the body.

**Figure 3:**
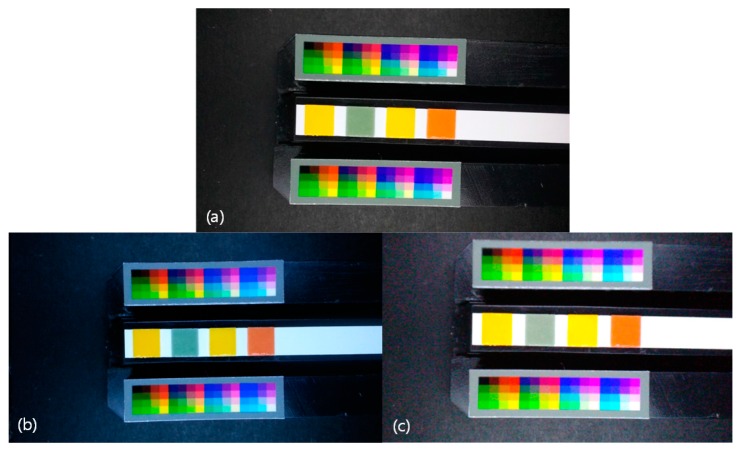
Captured images of pH 7.0 strip from three different smartphones. (**a**–**c**) corresponds to images from smartphone A~C, respectively.

In this experiment, three android smartphones named A, B, and C were used. Since the image is captured in the 3D printed mini light box, there are no side-effects caused by ambient light conditions. The mini light box eliminates the variation in lighting conditions [10]. Figure 3 shows the captured images of pH 7.0 strip. However, the locations of color patches on the reference regions and the target region are not in the fixed positions. Since the different camera characteristics and small movements of the smartphone cause the different color locations, it is required to apply the automatic color retrieving algorithm.

**Figure 4:**
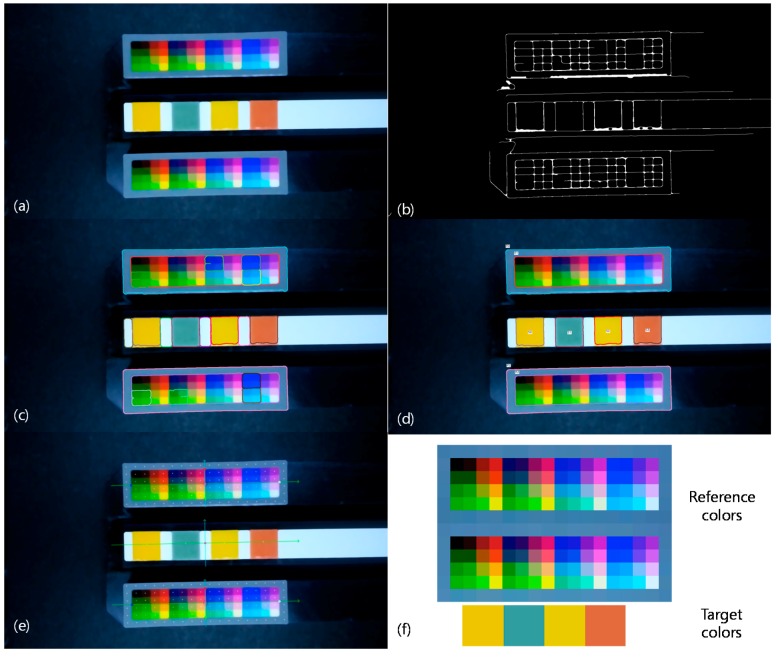
Automatic color retrieving algorithm: (**a**) Noise reduction and image simplification; (**b**) Edge detection; (**c**) Initial contour extraction; (**d**) Final contour labeling; (**e**) Automatic position determination to obtain reference colors and target colors; (**f**) Retrieved reference colors and target colors.

Figure 4 shows the step-by-step result of the automatic color retrieving algorithm. The six steps of the automatic color retrieving algorithm are illustrated from Figure 4a–f in consecutive order. In the noise reduction and image simplification step, a simplified image is obtained from the input capture image by applying the media filter [25]. The filtering strength is adjusted empirically. In the edge detection step, Canny edge detector [26] and morphological filtering are used to detect the final edge information. In the initial contour extraction step, only rectangular contours are collected. However, unwanted rectangular contours are still remained and the contours on the reference region are not separated from those on the target region. In the final contour labeling step, additional constraints are applied to separate the contours on the reference region from those on the target region. The following step is to determine the positions for retrieving reference colors and target colors automatically. Principal component analysis results performed on the reference region contours play an important role to compensate for the small movements of the smartphones. In the following final step, the reference colors and target colors are retrieved from the automatically determined positions. 

### 3.2. Calibration Step Results

**Figure 5:**
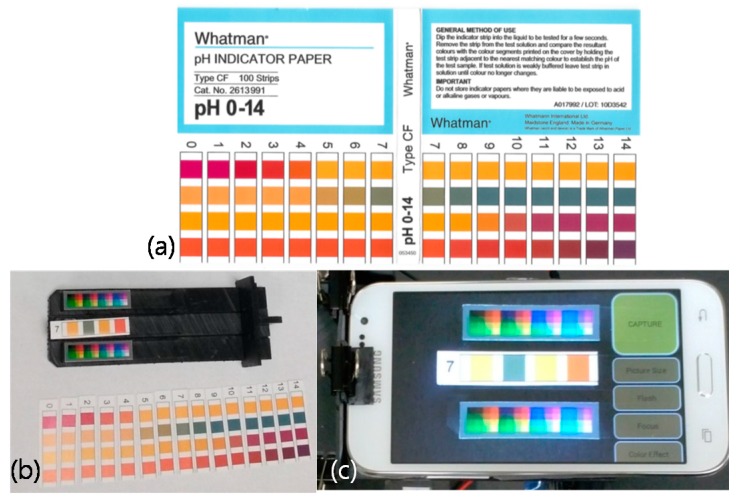
Preparations for calibration step: (**a**) Paper-printed comparison chart; (**b**) Split pieces; (**c**) Capturing the pieces on the mini light box.

Figure 5 shows the calibration preparation step based on the paper-printed comparison chart and the reference image. First, we split the comparison chart into 15 pieces according to the pH value. Then, we placed the piece into the light box and captured the image with the smartphone camera. According to the pH value, 15 images are acquired and used for calibration. In Figure 5, it should be noted that two reference images and a piece of comparison chart image are placed on the bottom_base and the bottom_stripbed, respectively.

**Figure 6:**
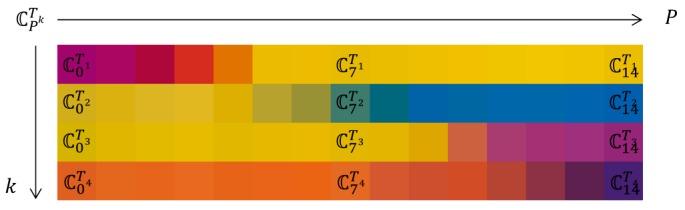
Calibrated target colors ${\mathbb{C}}_{P}^{T_{k}}$ obtained from all paper-printed pH stripes for all pH indexes.

The reference colors retrieved in the capture image may change for various reasons such as the built-in unknown automatic image correction operations of the smartphones. The calibration step uses the color adaptation algorithm to compensate for the unwanted changes. After completing the calibration step, the calibrated target colors ${\mathbb{C}}_{P}^{T_{k}}$ are obtained. Figure 6 shows the calibrated target color ${\mathbb{C}}_{P}^{T_{k}}$ obtained from all paper-printed pH stripes of the paper-printed comparison chart.

**Figure 7:**
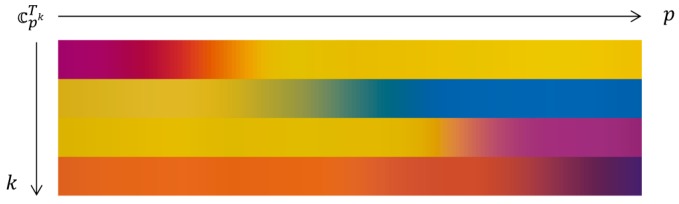
High-resolution calibrated target colors ${𝕔}_{p}^{T_{k}}\;$interpolated from ${\mathbb{C}}_{P}^{T_{k}}$.

Since we used the pH indicator paper of 1.0 pH resolution, the calibrated target color ${\mathbb{C}}_{P}^{T_{k}}$ also has a resolution of 1.0. However, in Figure 6, it is noticeable that there are no abrupt color changes as the pH value changes and the resolution can be improved by a sophisticated interpolation algorithm. In order to obtain higher resolution color information, we applied the non-rigid u’v’ curve interpolation based on thin plate spline interpolation. Figure 7 shows the obtained high-resolution calibrated target colors ${𝕔}_{p}^{T_{k}}$. In this paper, the pH resolution of 0.1 is used from pH ≈ 0.0 to pH ≈ 14.0 for ${𝕔}_{p}^{T_{k}}$. Although the color calibration is performed on the u’ and v’ components only, Figure 6 and Figure 7 are illustrated with RGB colors for better understanding. Compared with Figure 6, Figure 7 shows more continuous high-resolution color information.

**Figure 8:**
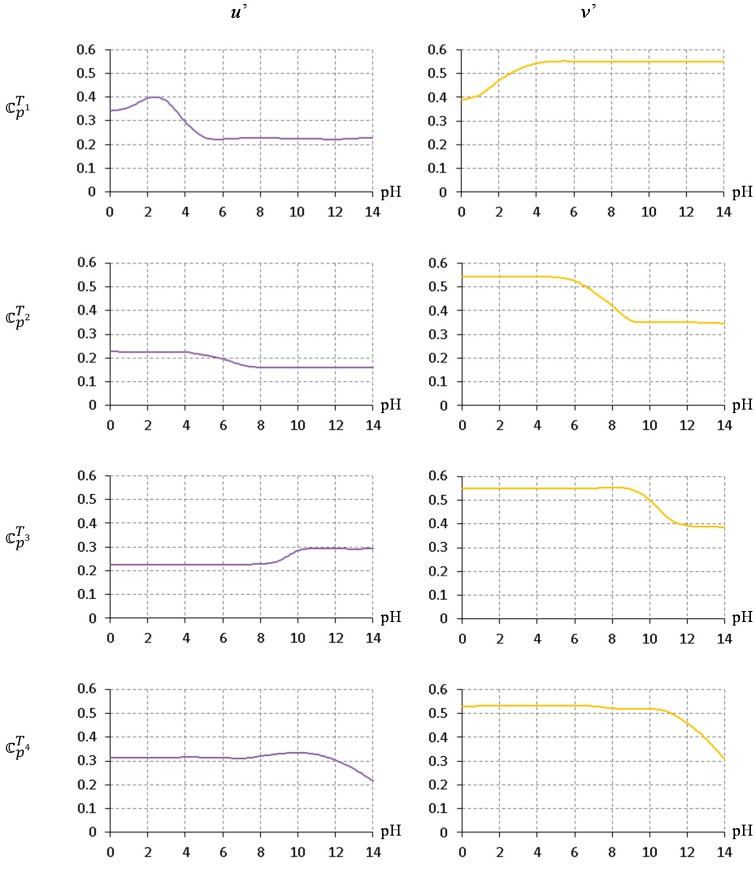
CIE 1976 u’ v’ color component of the high-resolution calibrated target colors ${𝕔}_{p}^{T_{k}}$.

Figure 8 shows the u’v’ color component of ${𝕔}_{p}^{T_{k}}$ corresponding to Figure 7. The curves of the 4 color patches are non-linear; therefore, finding the best match of $M^{T_{k}}$ among ${𝕔}_{p}^{T_{k}}$ can be a good solution to obtain the final pH estimate because it doesn’t require any prior knowledge of best-fitting curves for multiple color patches.

### 3.3. Measurement Step Results

**Figure 9:**
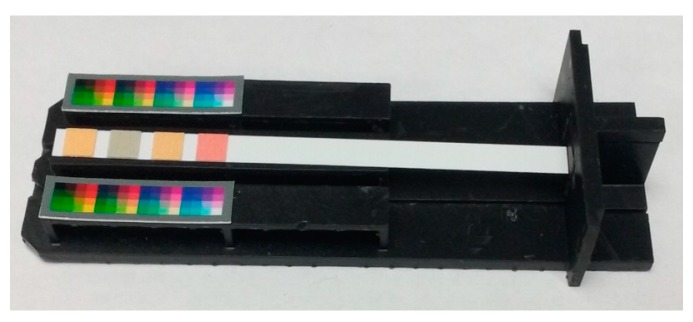
The bottom_base and the bottom_stripbed used in the measurement step.

In measurement step, a real pH indicator paper to measure is placed on the bottom_stripbed as shown in Figure 9. 

**Figure 10:**
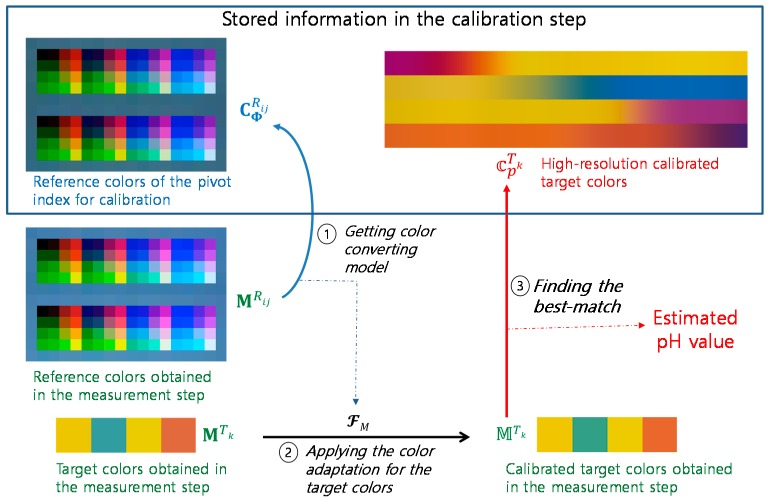
Overall procedure performed in the measurement step.

Figure 10 shows the overall procedure performed in the measurement step. It is assumed that the calibration data, $C_{\Phi }^{R_{ij}}$ and ${𝕔}_{p}^{T_{k}}$, are evaluated and stored in the calibration step. First, the color converting model $F_{M}$ is estimated from the color adaptation algorithm between $M^{R_{ij}}$ and $C_{\Phi }^{R_{ij}}$. Then, the calibrated target color $M^{T_{k}}$ corrected from the initial target color $M^{T_{k}}$ is obtained using the same color converting model $F_{M}$. Finally, the pH value is estimated by finding the best match of $M^{T_{k}}$ among ${𝕔}_{p}^{T_{k}}$. All operations in the measurement step are performed automatically on each of the three Android smartphones.

Table 1 shows the pH estimation results for real samples of standard pH buffer solutions in terms of average absolute error and standard deviation. Orion pH buffer solutions of pH 1.68, 4.01, 7.00, 10.01, 12.46 were used for the experiment. The pH indicator paper was captured four times. Although we use the pH indicator paper with resolution of 1.0 and perform the calibration step with the paper-printed comparison chart supplied by the manufacturer, the average errors are below 0.25 which is better than the original resolution of the indicator paper.

We applied a similar experiment for a few other samples. Ricca pH 6.0 BS, EMS pH 6.5 BS, and Gibco pH 7.4 PBS were used for this experiment and the results were summarized in Table 2. For BS samples, the results are quite similar to Table 1. The pH 7.4 PBS sample showed a relatively large error. Since the colorimetric prediction depends only on the obtained color information and the estimated pH values of the PBS sample are consistent, it may be caused by an intrinsic property of the particular PBS sample affecting to the color response of the pH indicator paper. The standard deviation columns shown in Table 1 and Table 2 show the consistency of the pH detection.

Table 1 and Table 2 show reasonably good estimation results but there are some estimation errors. The errors can be attributed to a variety of reasons. The product of the spectral radiant power of the light source and the spectral reflectance of the surface is known to affect the color of the surface [27]. Therefore, the color accuracy of the paper-printed comparison chart provided by the manufacturer is not guaranteed and it can’t be verified if the light conditions are not the same. The mini light box with two LEDs is a very simple structure, so the light illumination cannot be guaranteed to be uniform throughout the entire internal area. In addition, the paper characteristics of a comparison chart are quite different from the fabric-like color patches of a real pH indicator strip. Since the smartphone camera and the pH indicator paper are placed manually with some restrictions, accurate fixed locations are not guaranteed for the color information retrieval. The specular regions of an image are often saturated and can cause problems with all subsequent image processing algorithms [28]. Similarly, saturated color information caused by automatic brightness control and white balancing of the smartphone camera can cause the degradation of color adaptation performance.

Despite of all the factors causing the estimation errors, the proposed algorithm provides reasonably good estimation results. The estimation performance is expected to improve if manufacturers provide accurate color comparison charts and smart color adaptation techniques are applied.

## 4. Conclusions

We have developed a smartphone-based colorimetric pH detection system for point-of-care applications. Since the color is determined by the light source and the image sensor, the proposed system deals with the changes of the ambient light and the image sensor characteristics by means of a 3D printed mini light box and color calibration. The ambient light effects are blocked with the 3D printed mini light box. The color adaptation based on the third-order polynomial model is performed in the CIE 1976 u’v’ color space by using the reference paper in order to stabilize the color variations caused by built-in unknown automatic image correction operations. The high-resolution pH estimation is performed by using the non-rigid u’v’ curve interpolation and the best-matching method to handle the smooth nonlinear curve properties of multiple color patches. Automatic color retrieving algorithm has been also developed to automatically detect the color information in the calibration and measurement steps. Although we use the pH indicator paper with resolution of 1.0 and perform the calibration step with the paper-printed comparison chart supplied by the manufacturer, the experimental results show that the proposed algorithm provides reasonably good estimation results. The smartphone-based colorimetric measurement based on paper-printed accurate color comparison charts and smart color adaptation techniques is expected to be applicable to various point-of-care applications such as colorimetric µPAD-based diagnostics and chemical composition monitoring. Future work will focus on optimizing the proposed method for a specific point-of-care application.

## Figures and Tables

**Table 1:** pH estimation results for pH buffer solutions (Orion) of pH 1.68, 4.01, 7.00, 10.01, and 12.46.

Smartphone	pH (Ideal)	Estimated pH (with Four Captures)				Avg. |Err.|	Std. Dev.
		1	2	3	4		
A	1.68	1.5	1.5	1.5	1.5	0.18	0.00
	4.01	4.1	4.1	4.2	4.2	0.14	0.05
	7.00	7.2	7.2	7.2	7.2	0.20	0.00
	10.01	9.9	9.9	9.9	9.9	0.11	0.00
	12.46	12.5	12.5	12.4	12.4	0.05	0.05
B	1.68	1.5	1.5	1.5	1.5	0.18	0.00
	4.01	4.1	4.1	4.2	4.4	0.19	0.12
	7.00	6.9	7.0	6.9	7.0	0.05	0.05
	10.01	9.8	9.8	9.9	9.9	0.16	0.05
	12.46	12.6	12.5	12.5	12.0	0.17	0.23
C	1.68	1.6	1.6	1.6	1.5	0.11	0.04
	4.01	4.3	4.3	4.1	4.2	0.22	0.08
	7.00	7.2	7.2	7.3	7.3	0.25	0.05
	10.01	10	9.8	9.7	9.8	0.19	0.11
	12.46	12.3	12.3	12.3	12.2	0.19	0.04

**Table 2:** pH estimation results for a few other BS and PBS samples. pH 6.0 BS (Ricca), pH 6.5 BS (EMS), and pH 7.4 PBS (Gibco) are used.

Smartphone	pH (Ideal)	Estimated pH (with Four Captures)				Avg. |Err.|	Std. Dev.
		1	2	3	4		
A	6.0 BS	6.1	6.1	6.1	6.1	0.10	0.00
	6.5 BS	6.6	6.6	6.6	6.6	0.10	0.00
	7.4 PBS	8.4	8.4	8.4	8.4	1.00	0.00
B	6.0 BS	6.0	6.1	6.1	6.1	0.07	0.04
	6.5 BS	6.5	6.5	6.4	6.3	0.08	0.08
	7.4 PBS	8.2	8.3	8.2	8.3	0.85	0.05
C	6.0 BS	6.2	6.1	6.1	6.1	0.13	0.04
	6.5 BS	6.7	6.6	6.7	6.7	0.18	0.04
	7.4 PBS	8.5	8.4	8.5	8.5	1.08	0.04
